# Postural Stabilization Strategies to Motor Contagion Induced by Action Observation Are Impaired in Parkinson’s Disease

**DOI:** 10.3389/fneur.2018.00105

**Published:** 2018-03-01

**Authors:** Elisa Pelosin, Ambra Bisio, Thierry Pozzo, Giovanna Lagravinese, Oscar Crisafulli, Roberta Marchese, Giovanni Abbruzzese, Laura Avanzino

**Affiliations:** ^1^Department of Neuroscience, Rehabilitation, Ophthalmology, Genetics and Maternal Child Health, University of Genoa, Genoa, Italy; ^2^Ospedale Policlinico San Martino, Istituto di Ricovero e Cura a Carattere Scientifico per l’Oncologia, Genoa, Italy; ^3^Department of Experimental Medicine, Section of Human Physiology, Centro Polifunzionale di Scienze Motorie, University of Genoa, Genoa, Italy; ^4^INSERM-U1093, CAPS, Campus Universitaire, UBFC, Dijon, France; ^5^Istituto Italiano di Tecnologia, Centro di Neurofisiologia Traslazionale, Ferrara, Italy

**Keywords:** Parkinson’s disease, action observation, motor contagion, postural stabilization strategies, biological motion, chameleon effect

## Abstract

Postural reactions can be influenced by concomitant tasks or different contexts and are modulated by a higher order motor control. Recent studies investigated postural changes determined by motor contagion induced by action observation (chameleon effect) showing that observing a model in postural disequilibrium induces an increase in healthy subjects’ body sway. Parkinson’s disease (PD) is associated with postural instability and impairments in cognitively controlled balance tasks. However, no studies investigated if viewing postural imbalance might influence postural stability in PD and if patients are able to inhibit a visual postural perturbation. In this study, an action observation paradigm for assessing postural reaction to motor contagion in PD subjects and healthy older adults was used. Postural stability changes were measured during the observation of a static stimulus (control condition) and during a point-light display of a gymnast balancing on a rope (biological stimulus). Our results showed that, during the observation of the biological stimulus, sway area and antero-posterior and medio-lateral displacements of center of pressure significantly increased only in PD participants, whereas correct stabilization reactions were present in elderly subjects. These results demonstrate that PD leads to a decreased capacity to control automatic imitative tendencies induced by motor contagion. This behavior could be the consequence either of an inability to inhibit automatic imitative tendencies or of the cognitive load requested by the task. Whatever the case, the issue about the ability to inhibit automatic imitative tendencies could be crucial for PD patients since it might increase falls risk and injuries.

## Introduction

Postural control is a sensorimotor process in which the central integration of visual, vestibular, and proprioceptive information conveys the current state of equilibrium to ensure ongoing regulation of motor commands appropriate to the sensory experience (1). Thus, when a destabilizing event occurs, the postural control system acts in order to prevent a significant loss of equilibrium with stabilization mechanisms, mostly controlled at a subcortical level (2, 3). For a long time, these postural reactions have been considered to be automatically controlled; however, several studies demonstrated that these responses can be influenced by concomitant tasks or different contexts and then need to be modulated by a higher order motor control (4, 5).

An extensive literature analyzed the modulation of postural responses depending on voluntary movements (e.g., reaching movements), external perturbations (e.g., backward surface translation or unexpected stance perturbations), and environmental changes (e.g., height exposure or sensory illusions), but only few studies investigated postural changes determined by motor contagion induced by action observation (6–9).

Motor contagion’s theory arises from the ideomotor principle (10) stating that the mere thought of an action or the observation of others performing an action translates into action execution. Later, this theory was supported by further evidence demonstrating that individual’s motor responses are automatically influenced by the observed actions (11–15) and that brain regions responsible of motor representation of those actions are spontaneously activated in the observer’s brain (motor resonance effect) (16). Interestingly, motor contagion effect was observed also when watching non-human biological actions (17).

Regarding postural responses induced by action observation, Tia et al. (7) recorded postural changes during visual animations of human imbalance in healthy subjects, while staying barefoot on a force platform. Precisely, in order to expedite the phenomenon of postural contagion with the observed action, subjects were requested to be as relaxed as possible and to focus on the display instead of their own body. The mere observation of a point-light displaying a human model in postural imbalance was able to increase the observers’ body sway, indicating that this visual stimulus triggered a postural contagion. The authors suggested that body oscillations generated by the visual perturbation could produce a prediction-error *via* forward connections, that in turn would render the sensory feedback transiently inefficient to postural stabilization, thus showing the so-called “chameleon effect” (i.e., a spontaneous imitation tendency) (11).

Diseases affecting cortico-basal ganglia circuits, such as Parkinson’s disease (PD), are associated with postural instability and impairments in cognitively controlled balance tasks (18, 19). As an example, patients with PD perform poorly in postural stability tasks while concurrently performing a cognitive task (dual task) (20, 21). However, no data are available about the efficiency of stabilization mechanisms needed for counteracting perturbations induced by viewing postural imbalance in PD. This is of relevance since action observation training is becoming extremely used in rehabilitation of PD even for postural and balance disorders [for review, see Ref. (22, 23)].

Here, we assessed whether and how subjects with PD react during the observation of visual animations showing human imbalance. Particularly, we aimed to study whether postural reactions to this visual perturbation are still preserved in patients with PD. To accomplish that, we recorded participants’ body sway, during a point-light display of a gymnast performing a highly unstable postural task (biological condition) and during the viewing of a static image (control condition). This procedure, previously used by Tia et al. (7), allowed us to explore postural inhibitory processes required to counteract motor contagion induced by spontaneous tendency for imitation.

## Materials and Methods

### Subjects

Fourteen patients with idiopathic PD (mean ± SD age: 73.15 ± 4.16 years; 6 males and 8 females), and 17 healthy age-matched elderly (ELD) (mean ± SD age: 72.47 ± 5.20 years, 7 males and 10 females) participated in this study.

Patients were referred from the Department of Neurosciences of the University of Genoa by a movement disorders neurologist who made the diagnosis of idiopathic PD (according to the United Kingdom Parkinson’s Disease Society Brain Bank criteria). The mean score of the MDS—Unified Parkinson Disease Rating Scale, part III (Italian version) (24) was 28.86 ± 6.16 (± SD) and the Hoehn and Yahr stage (25) was 2.32 ± 0.25 (mean ± SD). The illness duration of patients was 7.76 ± 3.52 years (mean ± SD). All PD patients enrolled were under treatment with levodopa and/or dopamine agonists. The experiment took place during the “on” state (approximately 1 h after taking their anti-parkinsonian medications) for two reasons: (i) to avoid “off” tremor and bradykinesia, which could interfere with the aim of this experiment and (ii) because, unlike the effects on voluntary control, the ability to change postural responses has been shown not to improve with medication in PD patients (26, 27). Details of demographic and clinical characteristics of participants are provided in Table 1.

**Table 1:** Demographics and clinical characteristics of PD and ELD participants.

ID #	ELD		PD		
	Age (yr)/gender	Education	Age (yr)/gender	Education	UPDRD-III score
1	65/F	13	67/M	13	26
2	79/M	5	70/M	18	22
3	66/F	13	75/F	13	20
4	79/F	5	75/F	8	21
5	74/M	13	69/F	13	35
6	68/F	18	79/M	5	33
7	77/M	10	78/F	5	31
8	78/M	10	74/M	13	29
9	68/F	13	71/M	8	25
10	73/M	18	66/F	18	26
11	68/F	18	79/F	17	28
12	70/M	10	75/M	10	41
13	78/F	11	74/F	13	37
14	76/F	18	72/F	17	30
15	72/M	13			
16	77/F	16			
17	64/F	15			

*ID #, identification number; ELD, elderly; PD, Parkinson’s disease; yr, years; M, male; F, female; UPDRS-III, Unified Parkinson’s Disease Rating Scale, Motor Section*.

Exclusion criteria for both the healthy participants and subjects with PD were the following: (i) Mini-Mental Status Examination score <24 points (28), (ii) presence of pain, orthopedic lower limbs conditions, or other conditions limiting independent stance, and (iii) previous diagnosis of peripheral nerve disorders or other neurological conditions known to affect touch, proprioception, and/or motor control. In addition, PD patients with freezing of gait symptom and Hoehn and Yahr stage > 3 were excluded from the enrollment. All participants provided informed consent prior to testing. Experimental procedures were approved by the Ethical Committee of the University of Genoa and were carried out in agreement with legal requirements and international norms (Declaration of Helsinki, 1964).

### Experimental Procedure

#### Apparatus

The biological stimulus of postural imbalance (a point-light video displaying a gymnast balancing on a rope) and the control stimulus (a white cross) projected during the experimental task were identical to those previously used by Tia et al. (7). Briefly, the stimuli were produced by recording a gymnast (equipped with 23 reflexive markers) while keeping his balance on a metallic rope. Movements were recorded with a motion capture system (ELITE, Bioengineering Technology and Systems, Milan, Italy). Signals were sampled at 100 Hz, and the displacements of the markers were smoothed with a Hanning filter for removing high-frequency noise (see Video S1 in Supplementary Material).

Both biological and control stimuli were rear-projected on 2 m × 2 m screen, by a video projector with a 1,024 × 768 spatial resolution and with a vertical refresh rate of 60 Hz. During the entire experiment, subjects stood barefoot on the force platform (AMTI mod. OR6) with their feet axes forming an angle of 30°, the heels 2 cm apart, and with their arms alongside their trunk. The correct position of participants’ feet was indicated by pre-marked lines on the platform. Stimuli projection and the force platform recording were controlled by means of two computers linked by a network. An *ad hoc* Matlab (Matworks, Inc.) software was set up to synchronize video projection with the postural data recordings.

#### Task

The aim of the task was to maintain a stable upright stance during the entire duration of the experiment. Precisely, subjects were instructed to carefully observe the video trying to stay as still as possible.

**Figure 1:**
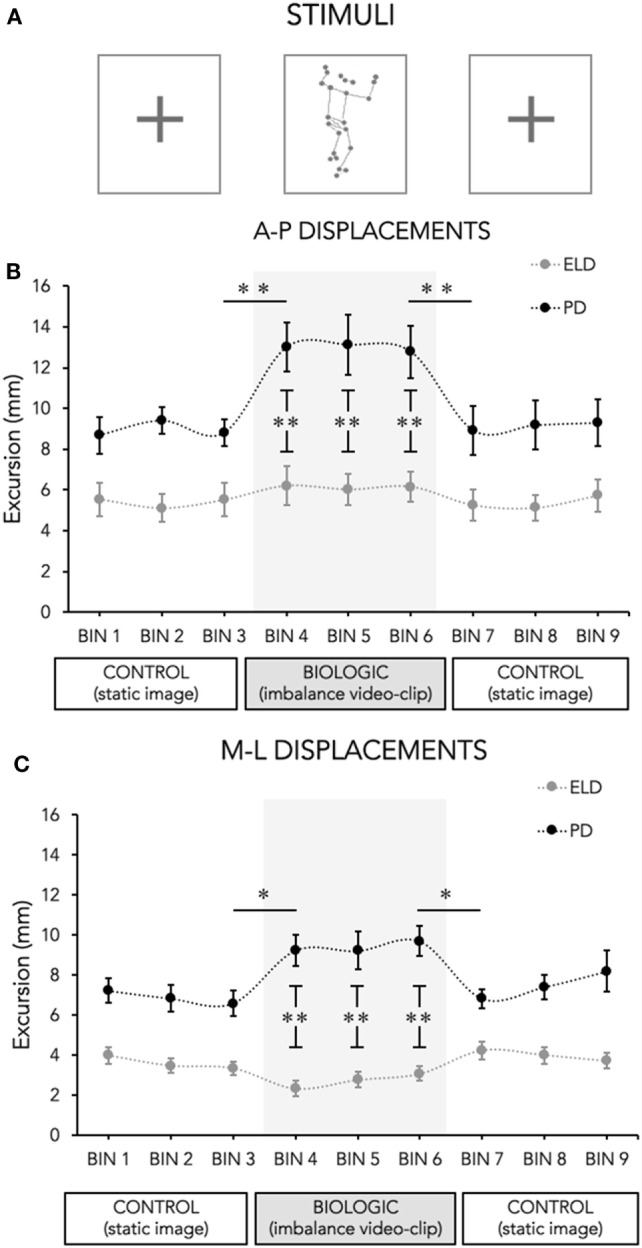
Mean of center of pressure (CoP) displacements in the two axes across time (Bin, 4 s each). **(A)** The different video displayed to the observer: Cross (Control stimulus) and a point-light video displaying a model in postural imbalance (biological stimulus). **(B)** r Mean of CoP displacements in antero-posterior (A-P) direction for Parkinson’s disease (PD, black circles) patients and for elderly (ELD, gray circles) during the experimental conditions. **(C)** Mean of CoP displacements in medio-lateral (M-L) direction for PD (black circles) and for ELD (gray circles) during the experimental conditions. The biological stimulus trials are highlighted by a gray box where asterisks refer to differences between groups. Bars indicate SDs; asterisks indicate statistical significant difference (^*^p < 0.05; **p < 0.001).

In order to investigate the effect of insertion and removal of the biological stimulus on postural stability, each experimental session was composed by (i) 12 s projection of the control stimulus (white cross), (ii) 12 s projection of a point-light displaying of a model in postural imbalance, and (iii) and again 12 s projection of the control stimulus (Figure 1A). This experimental session was repeated five times. The length of the video, corresponding to the duration of posturographic recording, was chosen to be short (12 s) in order to evaluate immediate postural reactions.

The entire experiment was performed in a quiet and dark room, free from any external distractions. At the end of the experimental session, we asked participants to answer the following questions: (1) if the point-light model was human or not; (2) what the model was doing; and (3) if they had perceived any postural perturbation. All participants’ answers were recorded by the investigator.

### Data Collection and Data Analysis

The center of pressure (CoP) is the point of location of the vertical ground reaction force vector and posturographic measures were derived from forces exerted against the ground during upright standing. During the acquisition, force platform signals were sampled at 40 Hz, amplified, and converted from analog to digital form. CoP displacements were recorded for the entire presentation of each stimulus and data were filtered using a second-order low-pass Butterworth filter (cutoff frequency 10 Hz). Coordinates for the antero-posterior (A-P, *y*-axis) and medio-lateral (M-L, *x*-axis) positions of the CoP were computed for postural sway assessment.

The following postural parameters were computed for each condition (control pre, biological, control post): (i) the CoP-length calculated as the excursions along the two axes (A-P and M-L) in the horizontal plane and (ii) the area encompassed by A-P and M-L displacements (sway area, computed as the surface of the confidence ellipse containing 95% of the CoP sampled positions).

For the analysis of the time course of CoP excursions on the A-P and M-L axes, we divided each 12 s trial into 4 s time bins and then postural parameters were calculated for each bin; the results were averaged across trials for each subject. We considered Bin 1, Bin 2, and Bin 3 the time bins of the control task (white cross) preceding the biological stimulus, Bin 4, Bin 5, and Bin 6 the time bins of the biological stimulus and Bin 7, Bin 8, and Bin 9 the time bins of the control task (white cross) displayed immediately after the biological stimulus. Similar to previous works (29, 30), the immediate effect of the visual perturbation was investigated by comparing data obtained from the time bin prior to and immediately after the insertion (i.e., Bin 3 vs Bin 4) and by comparing data obtained from the time bin prior to and immediately after the removal (i.e., Bin 6 vs Bin 7) of the biological stimuli, whereas the time course of postural control was determined by comparing postural parameters across Bin 4–Bin 6. For sway area, the grand average of the mean value obtained in the five recordings lasting 12 s of each condition (control pre, biological, control post) was used.

### Statistical Analysis

Chi-square test was applied to assess gender differences between groups. Prior to the analysis, all variables were examined for normality (Shapiro–Wilk *W* test) and mean and SD were calculated. Differences between groups (PD and ELD) for age an education were assessed by the non-parametric Mann–Whitney test. For the analysis of CoP A-P and M-L excursions, a repeated measures (RM) analysis of variance (ANOVA) was used with Group (PD, ELD) as between-subjects factor and Bins (Bins 3, 4, 5, 6, 7) as within-subjects factor. For the sway area, a RM-ANOVA with Group (PD, ELD) as between-subjects factor and TASK (Control-PRE, Biological, Control-POST) as within-subjects factor was performed. Finally, in order to assess whether possible changes induced by the observation of biological stimuli relied on postural performance recorded in control condition (PRE), a Pearson’s correlation analysis was performed.

The pre-defined level of significance was set at *p* < 0.05, with Bonferroni pairwise comparisons used as *post hoc* tests where appropriate.

## Results

Data from two participants (1 PD and 1 ELD) were excluded from the analysis because they reported having trouble to recognize the meaning of the biological stimuli. Thus, statistical analysis was performed on data obtained from 13 PD and 16 ELD subjects. No significant difference for sex, age, and education was found between participants enrolled in the two groups (Table 1). The mean values for each Bin of CoP excursions along the two axes (A-P and M-L) during the three phases of the experimental session are represented in Figures 1B,C, respectively.

### Participants’ Body Sway

Significant changes induced by the insertion or removal of the biological stimulus in A-P excursions were seen only in PD group (Figure 1B). Precisely, RM-ANOVA showed a significant main effect of Bins (*F*_4,108_ = 9.633; *p* < 0.001) and a significant Group × Bins interaction (*F*_4,108_ = 5.237; *p* = 0.001). *Post hoc* analysis showed a significant insertion (Bins 3 vs 4: *p* < 0.001) and removal effect (Bins 6 vs 7; *p* < 0.001) only in PD participants, whereas no changes where seen in ELD subjects (insertion: Bins 3 vs 4: *p* = 0,448 and removal: Bins 6 vs 7 *p* = 0.445). Additionally, *post hoc* analysis revealed that the increase of A-P excursions observed in PD participants immediately after the insertion of the biological stimuli persisted up to the end of the stimuli (Bins 3 vs 5: *p* < 0.001; Bins 3 vs 6: *p* < 0.001). Finally, when differences between groups were investigated (Figure 1B), *post hoc* analysis showed significant differences only during the imbalance stimuli (PD vs ELD: Bin 4, *p* = 0.001; Bin 5, *p* = 0.007, Bin 6, *p* = 0.002), whereas only a trend was found when comparing groups during the control stimuli (PD vs ELD: Bin 3, *p* = 0.083; Bin 7, *p* = 0.066).

For M-L excursions (Figure 1C), a significant Group × Bins interaction (*F*_4,108_ = 4.340; *p* = 0.003) was found, confirming that the observation of biological stimuli acted differently on the two groups. Indeed, *post hoc* analysis revealed that only in PD participants CoP excursions along M-L axis recorded in Bin 4 and Bin 6 were significantly larger than those recorded in Bin 3 and Bin 7, respectively (insertion Bins 3 vs 4: *p* = 0.031 and removal, Bins 6 vs 7: *p* = 0.028). No significant changes were detected in ELD subjects at the insertion (Bins 3 vs 4: *p* = 0.328) and at the removal (Bins 6 vs 7: *p* = 0.201) of biological stimuli. As for A-P excursions, data recorded across Bins 4–6 (Figure 1C, gray box) confirmed that the increase of M-L excursions in PD, lasted for the entire duration of biological tasks (Bins 3 vs 5: *p* < 0.024; Bins 3 vs 6: *p* < 0.010). Finally, *post hoc* analysis showed significant differences between the two groups (PD vs ELD: Bin 4, *p* < 0.001; Bin 5, *p* = 0.002, Bin 6, *p* < 0.001) during the imbalance stimuli only.

**Figure 2:**
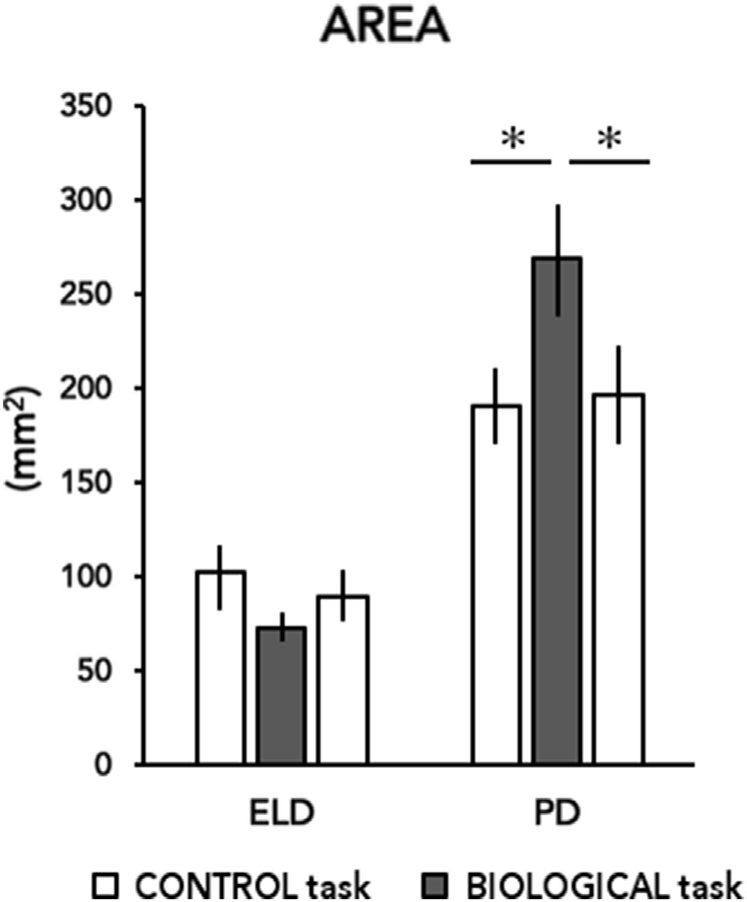
Area of center of pressure (CoP) excursions in the three stimulus conditions. The mean area of CoP displacement (computed as the surface of the confidence ellipse containing 95% of the CoP sampled positions) for each group (ELD, elderly and PD, Parkinson’s disease) during the experimental conditions (control and biological stimulus conditions) are shown. The biological stimulus trials are highlighted by a gray box where asterisks refer to differences between groups. Bars indicate SDs. Asterisks indicate statistical significant differences (^*^p < 0.05; **p < 0.001).

The mean value of the sway area for each group during the three stimulus conditions are depicted in Figure 2. RM-ANOVA showed a main effect of Group (*F*_2,54_ = 27.49; *p* < 0.001) and a significant interaction Group × Task (*F*_2,54_ = 6.99; *p* = 0.007). *Post hoc* analysis revealed significant changes in PD group (Control-1 vs Biological *p* = 0.005 and Biological vs Control-2 *p* = 0.031), whereas no changes were detected in ELD group (Control-1 vs Biological *p* = 0.245 and Biological vs Control-2 *p* = 0.531).

### Correlation Analysis of Postural Parameters

**Figure 3:**
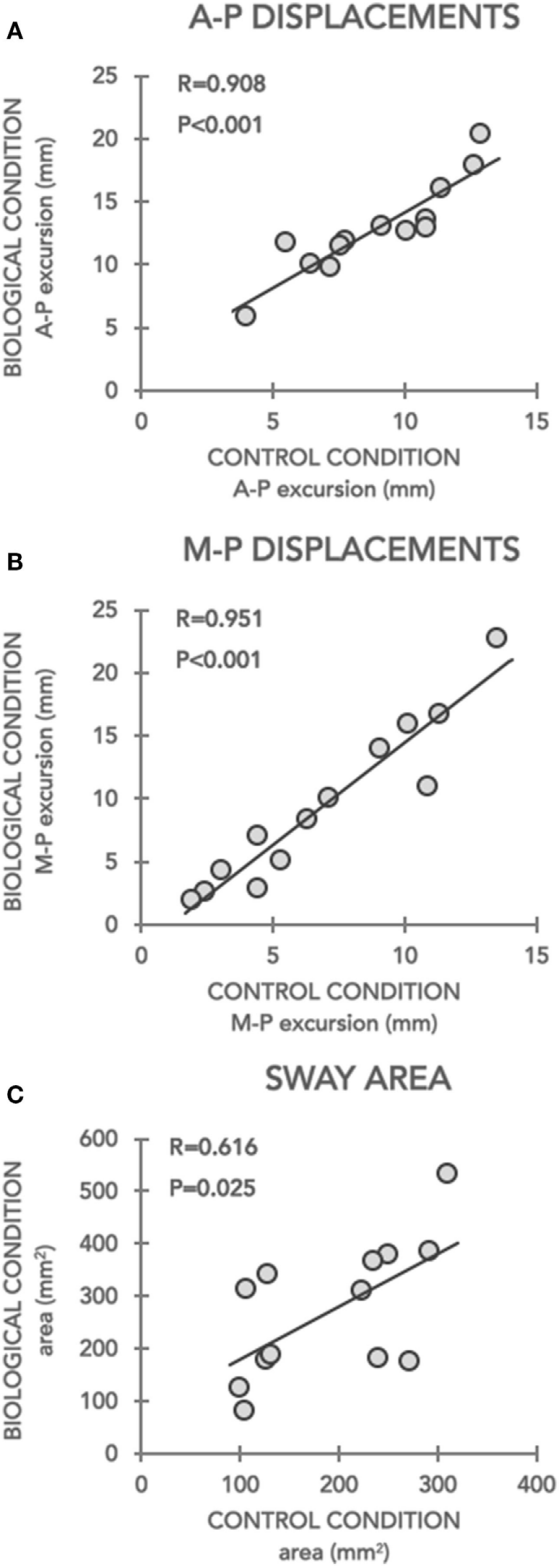
Correlations between postural parameters recorded during Control-PRE (*x*-axis) and during Biological conditions (*y*-axis). Correlations of data recorded in Parkinson’s disease subjects for antero-posterior (A–P) and medio-lateral (M–L) displacements and sway area are depicted in panels **(A–C)**, respectively. For A-P and M-L excursions, data are expressed in millimetres (mm), for sway area data are expressed in reported in squared-millimeters (mm^2^).

Linear relationships are shown in Figure 3. Postural parameters data recorded during all trials were averaged for Control-PRE (considered as baseline) and for Biological conditions, respectively, in each participant. Thus, the grand mean of A-P and M-L displacements and sway area data for Control-PRE and Biological conditions were used for running Pearson’s correlation analysis. Results showed that postural parameters recorded at the baseline (Control-PRE) significantly correlated with changes induced by imbalance stimuli in (i) A-P (*r* = 0.0908, *p* < 0.001; Figure 3A), (ii) M-L (*r* = 0.0951, *p* < 0.001, Figure 3B) displacements, and (iii) sway area (*r* = 0.616, *p* = 0.025; Figure 3C) only in patients with PD. Precisely, these relationships indicated that the larger were CoP excursions on the two axes and the area, recorded in the Control-PRE condition, the less was the ability to inhibit a visually induced postural perturbation.

### Participants’ Reports

Approximately 95% of participants (93% of PD and 94% of ELD) identified as a human stimulus the point-light video. Only, 1 out of 14 PD and 1 of 17 ELD subjects did not recognize that biological stimulus represented a human model, thus they were excluded from the study. As previously reported (7), also in our study, 100% of those subjects reported that the video was showing a subject in postural disequilibrium. Regarding perception of postural perturbation induced by biological stimuli, similarly to what observed by Tia et al. (7), around 40% of participants in both groups (6 out of 13 PD subjects and 6 out of 16 ELD subjects) reported having felt a slight increase of their body’s sway, with no difference between groups.

## Discussion

This study investigated the ability to inhibit a visually induced postural perturbation in a population of PD patients and in a control group of healthy age-matched subjects. The effect of action observation, in terms of motor contagion, on participants’ body sway has been addressed in previous studies (7, 8). However, to our knowledge, this is the first study investigating this phenomenon and the ability to counteract this imitative tendency in patients with PD. In summary, our results revealed a significant difference between the two groups in terms of postural control during the exposure to the biological stimuli. Indeed, a significant increase of sway area and CoP displacement (both on the M-L and A-P axes) was seen during the displaying of a model in postural imbalance only in PD participants. Precisely, this effect lasted for the entire duration of the biological stimulus and disappeared as soon as the control condition (white cross) was presented, so that body-sway values returned toward those recorded prior to the imbalance condition. Further postural parameters data at the baseline (Control-PRE) significantly correlated with changes induced by biological stimuli, suggesting that a reduced ability to inhibit postural contagion could be also partially related to postural instability in PD subjects.

Although we cannot directly affirm that a stabilization reaction occurred in healthy, since it would require a previous visual-induced postural imbalance, we might infer that it happened. Indeed, before starting the experiment, we required participants to remain as still as possible during the entire experimental session and all subjects in the ELD group were able to recognize that visual stimuli represented a human model in postural imbalance.

Our study demonstrates that, as shown by Tia et al. (7) in healthy subjects, it is possible to induce a postural contagion (chameleon-like mimicry effects) throughout the observation of stimuli displaying human imbalance also in patients with PD. Furthermore, in PD patients, the postural contagion affected postural control more widely than in healthy controls. Indeed, we adopted here the same biological stimulus used by Tia et al. (7). In our PD population, CoP excursions along both the A-P and the M-L axis were influenced by the observation of this biological stimulus. This result is not in accordance with what observed in the population of healthy subjects recruited by Tia et al. (7, 8). Indeed, in healthy, only A-P excursions were influenced by the observation of an upright point-light display of a gymnast balancing on a rope (7). This piece of evidence was further confirmed in another study showing that only when watching displays of A-P imbalance (and not M-L imbalance) greater area of CoP displacement was recorded from the observers, suggesting that postural contagion was promoted when the display was compatible with observers’ motor stabilization strategy, mainly oriented along the A-P axis (8).

Finally, we also found that PD leads to a decreased capacity to control automatic imitative tendencies, i.e., to adjust postural changes induced by visual motor contagion and that this ability correlated with baseline postural performance. Therefore, the issue about the ability to inhibit the automatic imitative tendencies in PD patients could be crucial.

### Imitation Tendency and PD

Chartrand and Bargh (11) defined the non-conscious mimicry of the postures, mannerisms, facial expressions, and other behaviors of one’s interaction partner, as the “chameleon effect.” Since then, most studies were focused on investigating mimicry during social interactions, showing that this spontaneous tendency of imitation plays an important role within social relationships, fosters liking between partners and increases empathy [for review, see Ref. (31)]. A large amount of neurophysiological and behavioral studies has revealed that the mere observation of actions leads to motor priming, triggers motor resonance processes that facilitate movement execution and induces a propensity to execute the observed actions (32–35). Precisely, the abovementioned tendency of imitation is automatic in that it occurs without will or awareness (12, 13, 36).

The discovery of mirror neurons system (MNS) in human cortex (37, 38) placed the neurophysiological bases for explaining the effects induced by action observation. Indeed, it has been shown that during the observation of actions executed by others, cortical areas congruent with those recruited during the execution of the observed action become automatically activated in the observer’s brain (39, 40). Further investigations revealed an involvement of the basal ganglia and cerebellum in action recognition and imitation processes, underlying that the mirror circuit does not work in isolation from subcortical structures (41–43). This might suggest that in PD patients, action observation abilities may be affected. However, neurophysiological data about the influence of PD pathology over the integrity of the MNS are still inconsistent. Reduced facilitation in action execution (44, 45), as well as reduced motor resonance (33), i.e., “the muscle-action specific facilitation of primary motor cortex during action observation,” and altered subthalamic activity during action observation have been reported in patients with PD (42, 46). In contrast, significant improvements of motor behaviors such as freezing of gait (47) or bradykinesia (48) and in disease severity (49) were also shown after one or repeated sessions of action observation.

Here, we found that the mere observation of point-light video displaying human imbalance elicited postural contagion, suggesting that imitation mechanisms through action observation are preserved in PD patients. Due to the complexity of this topic and differences among experimental designs used to explore imitation induced by action observation, we do not claim to have solved this issue. However, starting from recent evidences demonstrating that the recognition of biological motion triggers automatic imitation mechanisms in healthy (15, 17) and in neurological patients (50), we proved that through this protocol it is possible to induce motor contagion even in PD patients.

A recent EEG study investigated the cerebral dynamics of coding of postural control through action observation in healthy subjects (9). The authors demonstrated that while the observation of a person sustaining a quiet stance led primarily to the recruitment of temporal-parietal networks, the observation of a person in a condition of postural instability was coded in the central, but also in the parietal and temporal regions, slightly lateralized on the right hemisphere. Indeed, the superior temporal sulcus has been indicated by a large number of studies as the crucial node in the human MNS for detecting biological motion (51–54). Precisely, Martins et al. (9) described two specific cortical activity related to postural instability processing: a first activity recorded in the right temporoparietal regions (≈150 ms after the stimulus presentation) and a later activity (≈400 ms after the stimulus presentation) strictly lateralized in the occipital, temporal, parietal, and central electrodes of the right hemisphere. The authors proposed that the first activity might reflect the processing of the emotional load of the postural instability context associated to fear of falling, whereas the second activity indicates the right hemisphere as the “neural detector” for postural instability (55, 56).

Two hypotheses can be proposed to explain altered inhibition of postural imbalance induced by action observation in PD. The first is related to the possibility that in PD the neural structures responsible for the inhibition of imitative tendencies are malfunctioning, while the second takes into account an altered control of postural stabilization when cognitive load increases. Sensory deficits were unlikely to be responsible of postural contagion observed in PD patients. Indeed, participants’ reports about the ability to recognize as a human subject in postural disequilibrium the point-light video and about postural perturbation perception were similar between PD patients and healthy controls.

### Inhibition of Automatic Imitation of Postural Instability in PD

The control of automatic imitative tendencies has been extensively studied with neurophysiological and neuroimaging techniques. It has been shown that controlling imitative tendencies (i.e., avoiding unwanted imitation) occurs through the MNS modulation (57). Precisely, it was shown that motor resonance was greater during preparation to imitate than during preparation to counter-imitate a simple finger movement. These findings suggest the presence of some active inhibitory mechanisms required to control automatic imitation.

The candidate brain regions for controlling imitation have been indicated in the anterior medial prefrontal cortex and the right temporoparietal junction (TPJ). Indeed, lesions in these areas are associated with disruptive control of imitative tendencies (58, 59). Particularly, the right TPJ has been demonstrated to be involved in a specialized capacity to control automatic imitation of human agents (60), that is specific to self-other control (61), rather than to a universal process of conflict management. A recent resting-state fMRI study in PD (62) showed an altered functional integration, in terms of reduced node strength and betweenness centrality, in the dorsal anterior insula and TPJ nodes of the cingulo-opercular network. The authors discussed the disruptive functional integration of the TPJ node in the framework of the diverse functions carried out by this area, ranging from basic perception to social cognition. A paradoxical imitative facilitation has been demonstrated in PD subjects with voluntary self-initiation problems; for instance, difficulty in initiating gait after freezing, overcoming when another person or a cue drives the action (63) or difficulties in task-set maintenance reversed when there is a visual feedback or another person whose behavior they can imitate. The compromised TPJ function might also explain the lack of inhibition of imitation observed in the present study. Indeed, disrupting TPJ activity enhanced motor imitative ability, resulting in decreased control over imitation (64, 65).

However, this is only a tentative hypothesis since to address the neural basis of the control of imitative behavior during the observation of postural instability was beyond the scope of the present study and should be addressed in future studies.

### Postural Stabilization and Cognitive Load in PD

For a long time, postural reactions have been considered to be automatically controlled; however, it has been demonstrated that postural control is highly influenced by the amount of attention invested in keeping stance (66), by previous knowledge of an upcoming postural perturbation (67), and by the surrounding emotional context (68, 69). Therefore, postural control is now considered as a complex cognitive-motor function. As an example, when subjects have to control their posture during the execution of a mental task they must inevitably manage and allocate their attention both to postural control and to the other task simultaneously (70).

Postural instability is one of the major problems for individuals with PD, it affects simple everyday activities, and it increases the risk of falls (18), especially when PD patients have to adapt to mutable circumstances or environments. Although in PD subjects, falls were previously identified as a result of a pure motor problem (i.e., balance and gait dysfunctions), it is now accepted that falls are also related to cognitive function deficits (i.e., executive dysfunction). This is supported by the fact that dual and multitasking performance is severely compromised in PD, causing interference with attentional mechanisms that normally allow people to compensate for their balance and gait disturbances (19, 71, 72).

Considering the instruction given here to the participants (i.e., to carefully observe the video trying to stay as still as possible), changes in postural control seen during the observation of the biological stimuli could suggest the impairment of a cognitive-motor task. This hypothesis is supported by previous studies concerning the effect of a cognitive load on postural control in PD patients (18, 20, 73). In particular, alteration of CoP excursions during the execution of a secondary task has been found in PD, suggesting that patients may prioritize the “cognitive” task (“carefully observe the video”) over the primary motor task (“to stay as still as possible”). Furthermore, even if previous studies demonstrated that consciously focusing on postural balance may affect postural control (74, 75), recently Sciadas et al. (76) showed that attempting to reduce postural sway did not lead to changes in postural control in individuals with PD, under single task condition. Conversely, changes in CoP displacement and increase in M-L velocity were observed with the addition of a cognitive task, suggesting again a difficulty in prioritizing activities to maintain postural stability as tasks become more challenging. Finally, it is worthy to note that the biological stimulus and the control stimulus presented different degree of complexity (human dynamic figure vs white cross). This difference could also exert a potential influence on the cognitive loads of the two tasks, even if the great part of the cognitive load of the experimental condition (“carefully observe the video while staying as still as possible”) lies in the dynamic aspects of the biological stimulus. However, in a future study, this issue could be partly solved by using similar stimuli in both the experimental and control conditions (e.g., human dynamic figure vs static human figure).

## Conclusion

Our findings suggest that patients with PD are unable to stabilize postural control during the observation of a person in a condition of postural instability. This behavior could be the consequence either of an inability to inhibit automatic imitative tendencies or of the cognitive load requested by the task. We favor the first hypothesis since the cognitive load requested by the task was limited as indicated by the preserved ability to perform similar tasks in patients with cognitive deficits due to Alzheimer’s disease (77). However, since we did not insert a control condition displaying a “scrambled” animation, not a biological motion, but requiring attentional load (9), in our experimental protocol, future studies are needed to better discern between these two explanations. Further, we did not perform neuropsychological testing that could theoretically help in unrevealing the role of cognition in the ability to inhibit imitative tendencies in PD (78). A final limitation is related to the small sample size; to test this ability in a larger population will be warranted in future studies.

Whatever the case, we think that starting from the common use of action observation training as a rehabilitative strategy, this kind of impairment should be taken in account when planning balance rehabilitation programs for PD patients. Repeated observation of postural imbalance could be introduced as an observational training protocol to improve equilibrium control strategies and falls prevention in PD.

## Ethics Statement

This study was carried out in accordance with the recommendations of our institution. All subjects gave written informed consent in accordance with the Declaration of Helsinki. The protocol was approved by the local ethics committee (Comitato Etico Regione Liguria, IRCCS Azienda Ospedaliera Universitaria San Martino—IST, Genoa, Italy).

## Author Contributions

EP, AB, GL, and LA conceived and designed the experiments. GL, OC, and RM performed the experiments. EP, AB, and LA analyzed the data. EP, AB, OC, RM, GL, GA, and LA wrote the paper. EP, AB, TP, GA, and LA interpreted the data. EP, AB, TP, GA, and LA drafted the article. EP, TP, GA, and LA critically revised the article for important intellectual content.

## Conflict of Interest Statement

The authors declare that the research was conducted in the absence of any commercial or financial relationships that could be construed as a potential conflict of interest.

## Supplementary Material

Video S1 Sequence of frames of biological stimulus condition: a point-light video displaying a gymnast balancing on a rope.
